# Decreased plasma neuroactive amino acids and increased nitric oxide levels in melancholic major depressive disorder

**DOI:** 10.1186/1471-244X-14-123

**Published:** 2014-04-27

**Authors:** Yun-Rong Lu, Xin-Yan Fu, Li-Gen Shi, Yan Jiang, Juan-Li Wu, Xiao-Juan Weng, Zhao-Pin Wang, Xue-Yan Wu, Zheng Lin, Wei-Bo Liu, Hui-Chun Li, Jian-Hong Luo, Ai-Min Bao

**Affiliations:** 1 Department of Psychiatry, The Second Affiliated Hospital, Medical School of Zhejiang University, Hangzhou 310009, Zhejiang, P. R. China; 2 Department of Neurobiology; Key Laboratory of Medical Neurobiology of Ministry of Health of China; Zhejiang Province Key Laboratory of Neurobiology, Zhejiang University School of Medicine, 866 Yuhangtang Road, Hangzhou 310058, Zhejiang, P. R. China

**Keywords:** Major depressive disorder, Glutamic acid, Aspartic acid, Glycine, Gamma - aminobutyric acid, Nitric oxide

## Abstract

**Background:**

Amino acid neurotransmitters and nitric oxide (NO) are involved in the pathogenesis of major depressive disorder (MDD). Here we want to establish whether changes in their plasma levels may serve as biomarker for the melancholic subtype of this disorder.

**Methods:**

Plasma levels of glutamic acid (Glu), aspartic acid (Asp), glycine (Gly), gamma-aminobutyric acid (GABA), and NO were determined in 27 medicine-naïve melancholic MDD patients and 30 matched controls. Seven of the MDD patients participated also in a follow-up study after 2 months’ antidepressant treatment. The relationship between plasma and cerebral-spinal fluid (CSF) levels of these compounds was analyzed in an additional group of 10 non-depressed subjects.

**Results:**

The plasma levels of Asp, Gly and GABA were significantly lower whereas the NO levels were significantly higher in melancholic MDD patients, also after 2 months of fluoxetine treatment. In the additional 10 non-depressed subjects, no significant correlation was observed between plasma and CSF levels of these compounds.

**Conclusion:**

These data give the first indication that decreased plasma levels of Asp, Gly and GABA and increased NO levels may serve as a clinical trait-marker for melancholic MDD. The specificity and selectivity of this putative trait-marker has to be investigated in follow-up studies.

## Background

Amino acid neurotransmitters, i.e. the excitatory neurotransmitters glutamic acid (Glu) and aspartic acid (Asp), and the inhibitory neurotransmitters glycine (Gly) and gamma-aminobutyric acid (GABA), are involved in the pathogenesis of mood disorders such as major depressive disorder (MDD) [1,2]. The gaseous neurotransmitter nitric oxide (NO), which is synthesized from amino acid L-arginine (Arg) by NO synthases with citrulline (Cit) as a by-product also plays a significant role in depression [3]. MDD is thought to be due to the interaction between genetic, early developmental and environmental factors, leading to an individual’s stress response system becoming overly responsive to stressful life events, which causes this system to go into overdrive [4]. The hypothalamo-pituitary-adrenal (HPA)-axis, the monoamine systems and the autonomic nervous system are key regulating systems for stress responses and form major pathways for symptoms of depression [4,5]. Altered glutamatergic and reduced GABAergic and NO neurotransmission were observed in depression in a number of brain systems, which may significantly affect the neuronal activity involved in stress responses and mood regulation [1,2,6]. Changes in depression in the levels of these amino acids were found not only in different brain regions and cerebrospinal fluid (CSF), but also in blood and urine [7], which implies that they have the potential to serve as clinically relevant biomarkers or treatment efficacy monitors. Plasma levels of NO metabolites, i.e. nitrite and nitrate, which reflect the plasma NO concentrations, were also reported to increase in depression [8,9]. So far there are, however, quite some inconsistent findings concerning the plasma amino acid levels in depression. Decreased [10], increased [11], or no changes in plasma Gly [12] were reported in depression. In addition, no alteration [10] or increase in plasma Glu [11] was observed in depression. These controversial results may be due, at least partly, to the variety of depression subtypes studied, and to confounding factors such as different gender-ratios of samples, and/or different treatments.

Furthermore, amino acid transporter systems are located in the blood brain barrier (BBB) and transport amino acids from the blood into the brain and vice versa [13]. It is at present, however, not clear whether there is a relationship between the plasma and the CSF levels of these amino acids, although some researchers have proposed that the plasma levels of these neuroactive amino acids might, to a certain degree, reflect their brain levels [2,14].

The present study aimed, therefore, to analyze the plasma levels of 4 neuroactive amino acids, i.e. Asp, Glu, Gly and GABA, and NO levels calculated as Cit/Arg [15] in the major subgroup of melancholic MDD, medicine-naïve patients in their first depressive episode, in relation to their clinical symptoms. In addition, the relationship between plasma and CSF levels of these amino acids was analyzed in a group of non-depressive patients.

## Methods

### Subjects

Thirty Chinese Han race MDD patients and 30 age- and sex-matched control subjects had been recruited in the study by the Department of Psychiatry of the Second Affiliated Hospital of Zhejiang University School of Medicine. Three out of the 30 MDD patients appeared, however, later to develop bipolar disorder (BD). Therefore, 27 MDD patients (14 medicine-naïve males and 13 medicine-naïve females, age range 30 to 68 years) who were in their first depressive episode participated in the present study. They were diagnosed as melancholic MDD according to the Diagnostic and Statistical Manual of Mental Disorders, 4^th^ edition (DSM-IV) by qualified psychiatrists. The Mini International Neuropsychiatric Interview (Chinese modified version) was used to confirm the DSM-IV diagnosis. The 24-item Hamilton Depression Scale (HAMD) was used to rate the severity of depression, and a score of 35 or above was considered to be severe depression [16]. Seven out of the 27 melancholic MDD patients had suicidal thoughts, 7 attempted suicide before they joined the study and 1 actually committed suicide and died after one month of the treatment. Twelve out of the 27 melancholic MDD patients had no suicidal thoughts. The exclusion criteria were, apart from BD, as follows: psychiatric co-morbidity in the form of substance abuse disorders, psychotic disorders, anxiety disorders or mental retardation, chronic physical illness, or abnormal body mass index (BMI ≤ 18 or BMI ≥ 25). The follow-up protocol was as follow: after 2 months of antidepressant treatment (fluoxetine 20-40 mg/day) the patients came back to the clinic and their symptoms and HAMD scores were evaluated again, together with their blood samples being taken. Seven (6 males and 1 female) out of the 27 melancholic MDD patients whose clinical symptoms had improved and whose HAMD scores were at that moment lower than 14 (data not shown) voluntarily joined the follow-up study. The 30 healthy control subjects (15 males and 15 females, age range 30 to 65 years) who underwent their yearly physical examination in the same hospital voluntarily participated in this study during the same period. The exclusion criteria for the controls were as follows: abnormal BMI, medication for chronic illnesses or mental disorders.

Ten additional patients (4 males and 6 females, age range 20 to 68 years) with a range of medical conditions, including stroke, uterus with scar, leukemia, brainstem hemorrhage, and subarachnoid haemorrhage, and whose CSF and plasma samples were obtained for clinical reasons within the same hour, voluntarily participated in the present study during their stay in the same hospital. They were all during their clinical recovery phase. The inclusion criteria for this group were as follows: colorless, transparent CSF, leukocyte cell count < 100 × 10^6^/L, red cell count < 100 × 10^6^/L. The exclusion criteria for this group were as follows: abnormal BMI and/or psychiatric disorders.

The investigation was carried out in accordance with the latest version of the Declaration of Helsinki. All subjects signed informed consent forms and the study was approved by the Medical Ethics Committee of the Second Affiliated Hospital of Zhejiang University School of Medicine. Venous blood samples or CSF samples were collected between 6:00 a.m. and 9:00 a.m., and frozen at -80°C until measured.

### High performance liquid chromatography (HPLC) with fluorescence detection (FLD) for plasma and CSF amino acid analysis

The plasma (1 ml) and CSF (1-1.5 ml) samples were deproteinized with acetonitrile (1:1 v/v), lyophilized and diluted with 60% (v/v) methanol before analysis.

The plasma or CSF amino acid levels were determined by HPLC, which included pre-column derivatization with *o*-phthalaldehyde (Cat: O120, Pickering Laboratories, Inc. USA) and 3-mercaptopropionic acid (MPA, CAS: 107-96-0, Acros Organics, NJ, USA), reverse phase separation with Agilent 1100 series HPLC system and FLD (Agilent Technologies, Santa Clara, CA, USA). Two mobile phases were applied to the detection. The linearity of the detector response to standards was in the range of 13.2-79.0 μmol/L (Asp), 10.2-1447.0 μmol/L (Glu), 85.7-1000.0 μmol/L (Gly), 0.3-142.3 μmol/L (GABA), 0.9-158.2 μmol/L (Cit) and 1.8-178.0 μmol/L (Arg). The intraday relative standard deviations for the peak area were as follows: Asp, 2.27%; Glu, 2.14%; Gly, 1.59%; GABA, 3.89%, Cit, 0.97%; Arg, 0.77%, respectively. The interday relative standard deviations for the peak area were as follows: Asp, 2.69%; Glu, 6.37%; Gly, 4.29%; GABA, 6.62%, Cit, 4.29%; Arg, 3.18%, respectively.

### Statistical analysis

As the data were found to be not always normally distributed, non-parametric tests were applied. Comparison among 3 groups was performed by Kruskal-Wallis *H* test. Comparison between 2 groups was performed by Mann-Whitney *U* test. Comparison between 2 related samples was analyzed by Wilcoxon test. Correlations were examined with the Spearman test. *P* < 0.05 was considered to be significant.

## Results

**Figure 1:**
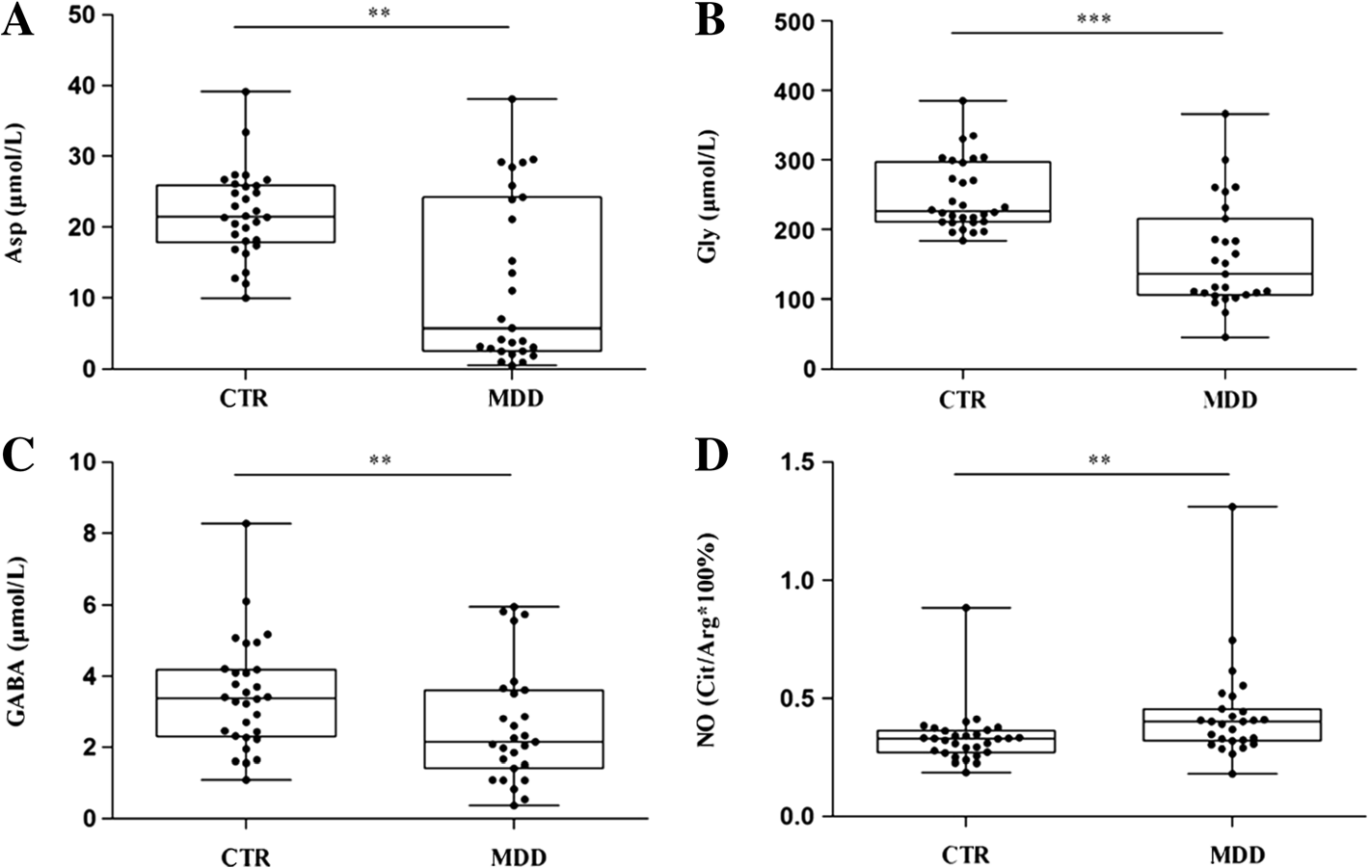
**Changes of plasma levels of neuroactive amino acids, and nitric oxide (NO, calculated as citruline/arginine) in melancholic major depressive disorder.** Boxplot showing the median, 25th-75th percentiles, and maximum and minimum value range. **(A)**: Asp, aspartic acid; **(B)**: Gly, glycine; **(C)**: GABA, gamma-aminobutyric acid; **(D)**: NO, nitric oxide. CTR, control; MDD, major depressive disorder. **P <0.01, ***P <0.001.

**Figure 2:**
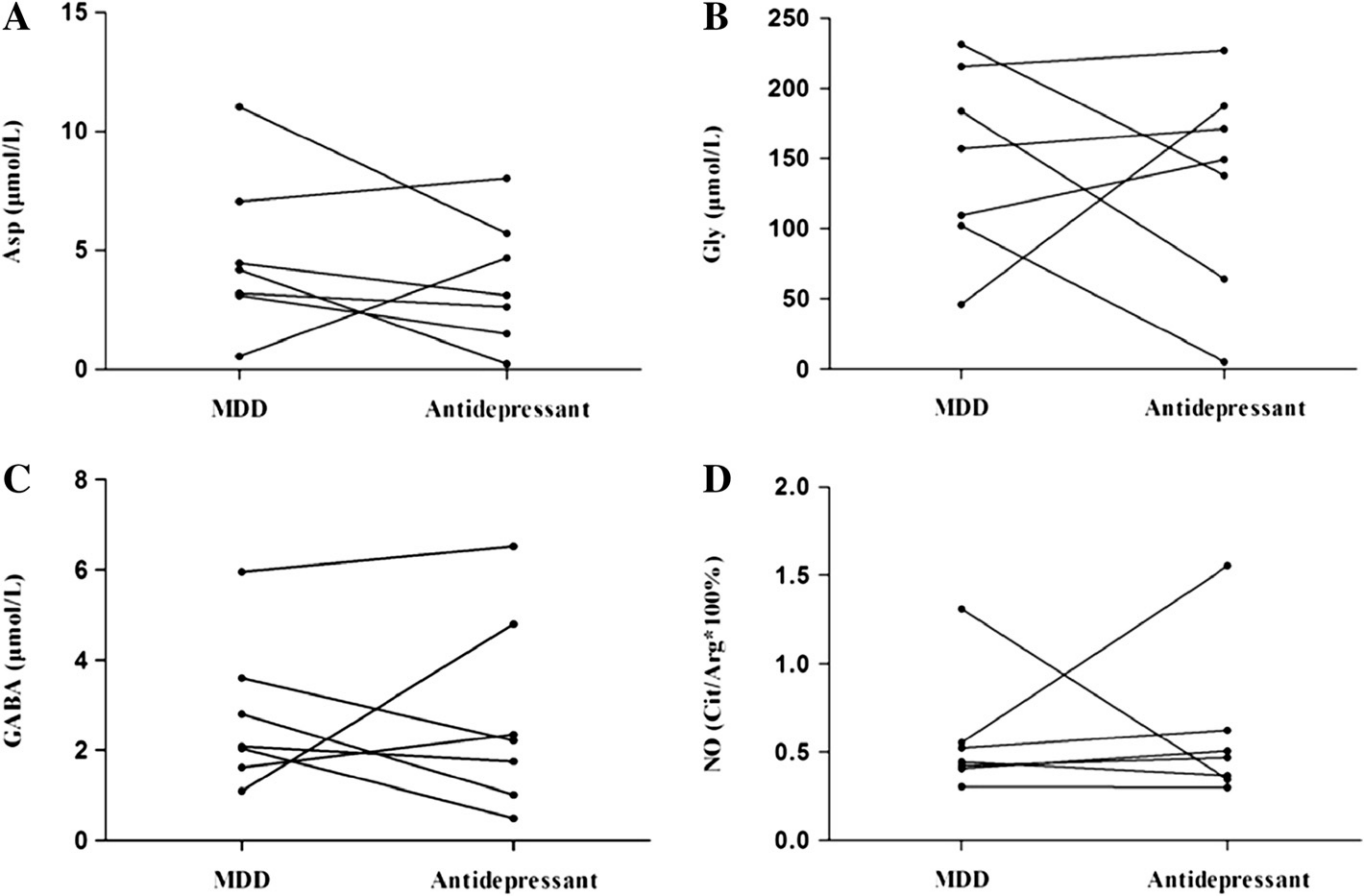
**Plasma levels of neuroactive amino acids and nitric oxide (NO) in melancholic major depressive disorder before and after 2 months of fluoxetine antidepressant treatment (20-40 mg/day). (A)**: Asp, aspartic acid; **(B)**: Gly, glycine; **(C)**: GABA, gamma-aminobutyric acid; **(D)**: NO, nitric oxide. MDD, major depressive disorder. Please note that there is no change in spite of the treatment (*P* ≥ 0.128).

The melancholic MDD group showed significantly decreased levels of plasma Asp (73.1%), Gly (39.9%), and GABA (36.6%), and significantly increased levels of NO (21.2%) compared with the control group (*P* = 0.003, *P* < 0.001, *P* = 0.021, and *P* = 0.005, respectively; Figure 1). No changes were found in plasma Glu levels (*P* = 0.646). In addition, there were significant positive correlations between Glu and Asp, and between GABA and Gly (*rho* = 0.756, *P* = 0.000; and *rho* = 0.479, *P* = 0.011; respectively), and a significant negative correlation between NO and Asp (*rho* = - 0.453, *P* = 0.018) in these MDD patients. When the relationships of the plasma levels of these 4 amino acids or NO levels and the different sub-phenotypes of the 9 symptoms of MDD according to the DSM-IV were checked (Table 1), it was observed that MDD patients who lost interest/pleasure or who had suicide thoughts had trends for higher plasma GABA levels (*P* = 0.076, and *P* = 0.054, respectively). The HAMD scores showed no significant correlations with these amino acid nor with the NO plasma levels (*P* ≥ 0.137). There were also no significant differences for the amino acid or NO levels between those with HAMD scores above 35 and those with HAMD scores below 35 (*P* ≥ 0.246). Moreover, for the 7 MDD patients who participated in the follow-up test, no significant differences were found for these amino acids or NO plasma levels before and after fluoxetine treatment (*P* ≥ 0.128, Figure 2).

**Table 1:** Comparison of plasma amino acids levels and nitric oxide levels in the 9 symptoms of major depressive disorder according to the DSM-IV

**Symptom items**	**Subgrouping**	**Asp**	**Glu**	**Gly**	**GABA**	**NO**
Item 1: depressed mood	No: n = 0	∕	∕	∕	∕	∕
	Yes: n = 27					
Item 2: diminished interest	No: n = 3	0.643	0.841	0.700	0.076	0.817
	Yes: n = 24					
Item 3: weight loss	No: n = 13	0.382	0.898	0.438	0.645	0.382
	Weight gain: n = 0					
	Weight loss: n = 14					
Item 4: disordered psychomotor state	No change: n = 4	0.417	0.450	0.883	0.985	0.346
	Agitation: n = 10					
	Retardation: n = 13					
Item 5: disordered sleep	No: n = 1	0.847	0.616	0.773	0.312	0.211
	Hypersomnia: n = 2					
	Insomnia: n = 24					
Item 6: fatigue or loss of energy	No: n = 9	0.382	0.647	0.643	0.396	0.607
	Yes: n = 18					
Item 7: feeling of worthlessness or guilt	No: n = 11	0.374	0.775	0.402	0.711	0.693
	Yes: n = 16					
Item 8: diminished ability to think or concentrate, or indecisiveness	No: n = 9	0.837	0.617	0.719	0.662	0.090
	Yes: n = 18					
Item 9: thought of death or suicide	No: n = 12	0.118	0.504	0.495	0.054	0.558
	Yes: n = 15					

**Notes:** Comparison of plasma levels of the 4 amino acids or NO levels among different sub-phenotype groups, based upon the 9 symptoms of major depressive disorder according to the DSM-IV. No: without this symptom; Yes: with this symptom. The numbers in the Table are P values. Comparison among 3 subgroups was performed by Kruskal -Wallis *H* test. Comparison between 2 subgroups was performed by Mann-Whitney *U* test. Asp: aspartic acid; Glu: glutamic acid; Gly: glycine; GABA: gamma-aminobutyric acid; NO: nitric oxide. The 9 symptoms includes: 1. depressed mood most of the day, nearly every day, as indicated by either subjective report (e.g. feels sad or empty) or observation made by others (e.g. appears tearful); 2. markedly diminished interest or pleasure in all, or almost all, activities most of the day, nearly every day (as indicated by either subjects account or observation made by others); 3. significant weight loss when not dieting or weight gain (e.g. a change of more than 5% of body weight in a month), or decrease or increase in appetite nearly every day; 4. psychomotor agitation or retardation nearly every day (observable by others, not merely subjective feelings of restless or being showed down); 5. insomnia or hypersomnia nearly every day; 6. fatigue or loss of energy nearly every day; 7. feelings of worthlessness or excessive or inappropriate guilt (which may be delusional) near every day (not merely self-reproach or guilt about being sick); 8. diminished ability to think or concentrate, or indecisiveness, nearly every day (either by subjective account or as observed by others); 9. recurrent thoughts of death (not just fear of dying), recurrent suicidal ideation without a specific plan, or a suicide attempt or a specific plan for committing suicide.

The melancholic MDD patients showed no sex differences in these amino acids or NO plasma levels (*P* ≥ 0.275). However, the subjects in the control group showed a significant sex difference in plasma Glu levels: male subjects had a significant 66.6% higher Glu level than female subjects (*P* = 0.003), which was not seen for Asp, Gly, GABA or NO levels (*P* ≥ 0.206). In addition, a significant positive correlation was observed between plasma levels of Asp and Glu, Asp and Gly, Asp and GABA, and Glu and GABA (*P* = 0.0001, *P* = 0.002, *P* = 0.017, and *P* = 0.041, respectively). No significant correlation was observed between plasma levels of these amino acids and NO (*P* ≥ 0.470).

In the additional 10 non-depressive patients who were during their clinical recovery phase no significant correlations were found between plasma and CSF levels of Asp, Gly, GABA and NO (*P* ≥ 0.511), although there was a trend towards a positive correlation between plasma and CSF levels of Glu (*rho* = 0.588, *P* = 0.074).

## Discussion

In the present study we determined the plasma levels of 4 neuroactive amino acids, i.e. Asp, Glu, Gly and GABA, and of NO levels in medicine-naïve melancholic MDD patients in their first depressive episode. Significantly lower levels of Asp, Gly and GABA, and significantly higher levels of NO, which endured after 2 months of fluoxetine treatment, were observed in these patients, indicating that they might serve as a trait-marker, rather than a state-marker, for melancholic MDD. The fact that none of these parameters showed a clear correlation with HAMD scores or with the severity of depression fits the idea of a potential trait-marker.

Our finding of decreased plasma GABA levels in melancholic MDD was consistent with previous reports, which, in general, showed a significant decrease in GABA levels in plasma, CSF and brain tissue (for review, see [2]). In the 7 melancholic MDD patients whose clinical symptoms improved and who had agreed on repeated measurement we did not find significant changes in the GABA levels after 2 months of fluoxetine treatment. These data are in agreement with previous studies reporting that the decreased plasma GABA levels in MDD patients were not affected by the mood states, the severity of depressive symptoms or the antidepressant treatment [2]. It is noteworthy that in one study electroconvulsive therapy (ECT) showed no significant effect on plasma Gly but did result in increased plasma Asp and decreased plasma GABA levels in depressed patients [17]. The fact that ECT may significantly change the permeability of the BBB [18] may explain these alterations. It was interesting to see, however, that when we subgrouped these melancholic MDD patients according to each of the 9 symptoms of MDD (Table 1), we observed that those who lost interest/pleasure or who had suicide thoughts had trends for higher plasma GABA levels, although the MDD patients as a whole group showed lower GABA levels than controls. This point has to be investigated further in the future in a larger cohort of patients.

To our knowledge, this is the first report of a significant decrease in plasma Asp levels in medicine-naïve melancholic MDD patients. Altamura et al. [10] reported that there was no significant alteration of plasma Asp levels in untreated depressed patients. It should be noted, however, that their patients included BD subjects, while there was a study showing a significant increase both in plasma Glu and Gly in BD patients during the manic phase [19]. Mauri et al. [12] also reported no significant changes in plasma Asp and Gly levels, but they studied non-melancholic MDD patients. That Mauri et al. did not find changes in the plasma amino acid levels after 2 months of fluoxetine treatment was, however, in agreement with our present study. Please note that the sex difference in plasma Glu levels may have contributed to the discrepancy with these studies since the samples involved contained different gender-ratios.

The present results of increased plasma NO levels in melancholic MDD patients are consistent with a number of previous reports [8,9], which also found elevated NO plasma levels in MDD patients. It should be noted that these reports indicated that increased plasma levels of NO might be accompanied with suicide attempts in these patients [8,9], which was not observed in the present study. Interestingly, we found a significant negative correlation between the plasma levels of NO and Asp in melancholic MDD, which is consistent with the findings that testosterone levels are changed in MDD [20], and that NO inhibits whereas Asp promotes testosterone levels [21,22].

Some putative causes of the observed changes in plasma levels of neuroactive amino acids and NO in melancholic MDD should be considered. The HPA-axis and the autonomic nervous system are activated in stress responses and in depression. It was found in rats that activation of the sympathetic nervous system may lead to decreases in plasma Glu levels [23] due to significant increases in 2 enzymes, i.e. glutamate pyruvate transaminase and glutamate-oxaloacetate transaminase, which may accelerate the conversion of Glu to 2-ketoglutarate [24]. In addition, the stress compounds corticotrophin-releasing hormone (CRH) and adrenaline have been demonstrated to significantly and consistently decrease blood Glu levels in rats [25]. Our previous studies have found indeed significantly higher plasma levels of CRH in melancholic MDD patients compared with healthy controls (unpublished data). Whether the hyperactive HPA-axis and the sympathetic nervous system are involved in the mechanism which decreases the plasma levels of the amino acids Asp, Gly and GABA warrants further study. It is also known that stress-related events, including depression, are characterized by modifications of oxidative/nitrosative pathways in the brain [26]. Considering the important role of NO in oxidative stress [27], our finding of significantly increased plasma NO levels appear to at least partially corroborate the stress theory of depression. Furthermore, these neuroactive amino acids are non-essential amino acids that the body could synthesize by itself. Melancholic MDD patients have a decreased appetite, and thus also decreased food intake, leading to a reduced metabolism and decreased synthesis of these amino acids. This is also the reason why we limited the range of BMIs in the present study. Finally, BBB permeability may be changed in pathological conditions, as it does in the case of vascular brain edema and brain tumors [13], thus affecting the transportation between the brain and the blood of amino acid neurotransmitters. However, such BBB changes have, as far as we know, not been reported in MDD.

The study on the relationship between plasma and CSF levels of amino acids was carried out in a group of non-depressed individuals with different diseases who were during their clinical recovery phase. It is a limitation of present study that, for ethical reasons, we could not study CSF from MDD patients or healthy control subjects. It cannot be excluded that the medical conditions from which these patients were recovering may have affected BBB function. It should be noted, however, that the absence of correlations that we found between plasma and CSF amino acid levels is in agreement with the report that there were no significant correlations between venous blood and CSF levels of Asp or Gly in healthy subjects [28]. This implies that the 2 compartments, i.e. the blood and the CSF, do not seem to have an equilibrium mechanism for the transportation of these amino acid neuroactive substances. Another limitation of the present study is that the study was based upon a relatively limited sample size and only 7 patients finished the follow-up study. It should be noted, however, that although the number of followed up patients is relatively small, the Wilcoxon test for these 7 patients, that showed significant clinical improvement after 2 months antidepressant treatment, did not show significant changes in these amino acids or NO plasma levels before and after treatment, which supports the suggestion that these parameters might serve as trait-markers for melancholic subtype of MDD.

## Conclusion

Melancholic MDD patients have significantly decreased plasma levels of Asp, Gly and GABA and increased NO levels. Larger patient groups and other types of MDD should be investigated before we can conclude that indeed the changes in the plasma neuroactive amino acids and NO are trait-, rather than state-, marker for melancholic MDD.

## Competing interests

The authors declare that they have no conflicts of interests.

## Authors’ contributions

Author YRL, XYF, YJ, JHL and AMB designed the study and wrote the protocol. Author ZL, WBL, and HCL strongly supported the clinical study. Author LGS, ZPW, JLW, XJW, XYW and AMB managed the literature searches and analyses. Authors YRL, XYF, LGS, ZPW and XYW undertook the statistical analysis, and author YRL, XYF, LGS and AMB wrote the first draft of the manuscript. All authors contributed to and have approved the final manuscript.

## Pre-publication history

The pre-publication history for this paper can be accessed here:

http://www.biomedcentral.com/1471-244X/14/123/prepub
